# Characterization of Domestication Loci Associated with Awn Development in Rice

**DOI:** 10.1186/s12284-022-00607-y

**Published:** 2022-11-30

**Authors:** Ngoc Ha Luong, Sangshetty G. Balkunde, Kyu-Chan Shim, Cheryl Adeva, Hyun-Sook Lee, Hyun-Jung Kim, Sang-Nag Ahn

**Affiliations:** 1 Department of Agronomy, College of Agriculture and Life Sciences, Chungnam National University, Daejeon, 34134 South Korea; 2 Department of Agronomy, ARS, Anniger Dist., Dharwad, Karnataka India; 3 Crop Breeding Division, National Institute of Crop Science, Wanju-Gun, 55365 South Korea; 4 LG Chem., Ltd., Seoul, 07796 Korea

**Keywords:** Quantitative trait loci, Awn development, *qAwn9*, Rice, *O. minuta*

## Abstract

Rice (*Oryza sativa* L.) is a widely studied domesticated model plant. Seed awning is an unfavorable trait during rice harvesting and processing. Hence, loss of awn was one of the target characters selected during domestication. However, the genetic mechanisms underlying awn development in rice are not well understood. In this study, we analyzed and characterized the genes for awn development using a mapping population derived from a cross between the Korean *indica* cultivar ‘Milyang23’ and a near-isogenic line NIL4/9 derived from a cross between ‘Hwaseong’ and *Oryza minuta*. Two quantitative trait loci (QTLs), *qAwn4* and *qAwn9*, mapped on chromosomes 4 and 9, respectively, increased awn length in an additive manner. Through comparative sequencing analyses of the parental lines, *LABA1* was determined as the causal gene underlying *qAwn4. qAwn9* was mapped to a 199-kb physical region between markers RM24663 and RM24679. Within this interval, 27 annotated genes were identified, and five genes, including a basic leucine zipper transcription factor 76 (*OsbZIP76*), were considered as candidate genes for *qAwn9* based on their functional annotations and sequence variations. Haplotype analysis using the candidate gene revealed tropical-*japonica* specific sequence variants in the *qAwn9* region, which partly explains the non-detection of *qAwn9* in previous studies that used progenies from interspecific crosses. This provides further evidence that *OsbZIP76* is possibly a causal gene for *qAwn9*. The *O. minuta qAwn9* allele was identified as a major QTL, providing an important molecular target for understanding the genetic control of awn development in rice. Our results lay the foundation for further cloning of the awn gene underlying *qAwn9*.

**Supplementary Information:**

The online version contains supplementary material available at 10.1186/s12284-022-00607-y.

## Background

Cereal crops have been the principal component of human diets for thousands of years, with the most highly productive crops (rice, wheat, and maize) contributing to more than half of the calories consumed (Awika 2011). Despite the critical importance of these crops, their history of cultivation is poorly understood, and their origin is still debated even after intensive studies (Doebley et al. 2006; Sweeney and McCouch 2007). Domestication was conducted through selection driven by human that considerably changed the morpho-physiological characteristics of wild species to facilitate efficient farming, harvesting, and consumption (Tang et al. 2010). To satisfy the growing human demand, some important traits, such as easy harvest, high yield, and low toxicity, are preferred during the selection process. The presence of awns, an unfavorable characteristic during harvest and processing, has been targeted by selection programs (Ishii and Ishikawa 2018). The loss of awn in some cereals, including cultivated rice (*Oryza sativa* L.), is a prominent example of domestication (Hua et al. 2015). Plant adaptation has facilitated the presence of awns in many wild rice species to protect against animal predation and to facilitate the motility required for seed dispersal (Elbaum et al. 2007; Yoshioka et al. 2017). In contrast, through artificial selection and breeding programs, rice domestication has led to cultivated rice with shorter, or even without, awns that facilitate seed collection, storage, and processing by humans.

Considerable interest exists in discovering genes and genetic mechanisms contributing to awn reduction during domestication because it may facilitate basic research and trait manipulation through breeding strategies (Bessho-Uehara et al. 2021; Ross-Ibarra et al. 2007). Moreover, rice domestication initially resulted from selection on natural mutations in wild rice. Therefore, identifying domestication genes using interspecific crossing is an effective and accurate way to provide sufficient evidence to understand the rice domestication process. Additionally, wild rice species are not only important resources for rice breeding but also provide a potential opportunity for identifying novel genes (Solis et al. 2020).

Recently, studies have been conducted to elucidate the genetic mechanisms underlying the development of rice awns. These results suggest that awn development is a complex trait regulated by many genes and is mostly observed in wild rice (Bessho-Uehara et al. 2021; Amarasinghe et al. 2020; Qin et al. 2021; Wang et al. 2011). Although several awn-related quantitative trait loci (QTLs) have been detected, only a few major QTLs have been cloned and characterized at the molecular level. *An-1* encodes a basic helix–loop–helix (bHLH) transcription factor that regulates cell division, promotes awn development, and reduces the grain number (Luo et al. 2013). *An-2*/*LABA1* (*LABA1*) encodes a critical enzyme for cytokinin biosynthesis that promotes awn elongation by enhancing cell division and decreasing grain production, through reducing the number of grains per panicle and tillers per plant (Gu et al. 2015; Hua et al. 2015). *RAE2/GAD1/GLA* (*RAE2*) encodes a secreted peptide, and the loss of function of *RAE2* results in an increased number of grains per panicle, shorter grains, and an awnless phenotype (Bessho-Uehara et al. 2016; Jin et al. 2016; Zhang et al. 2019). The auxin response factor *OsETT2* and the *YABBY* transcription factor *DROOPING LEAF* (*DL*) are related to awn formation (Toriba and Hirano 2014). Although these studies have improved our understanding of the genetic basis of awn development, the molecular mechanism for the transformation of wild rice long awns to awnless cultivated rice remains poorly understood. Furthermore, the genes isolated to date have a substantial effect on awn development. These have created an intense desire to increase efforts to identify and clone the remaining genes with smaller effects.

Our previous study detected *qAwn9* for awn development near the simple sequence repeat (SSR) marker RM215 on chromosome 9 (Linh et al. 2006). However, phenotypic data of the F_2_ population suggested that at least two genes controlling awn length in the cross combination. It is impossible to detect other awn-related genes because this F_2_ population is segregated only in the region containing the *Oryza minuta* segment.

To continue our previous study, the objectives of this present study were: (1) to confirm and specify the target region containing QTL associated with awn length on chromosome 9 using a BC_1_F_2_ population derived from a new cross between the Korean *indica* cultivar ‘Milyang23’ and NIL4/9, (2) detect the other QTLs in addition to *qAwn9* that are associated with awn development in the population, (3) investigate the genetic interaction between the discovered QTLs, and (4) to determine the candidate gene for *qAwn9*.

## Materials and Methods

### Plant Materials

**Fig. 1:**
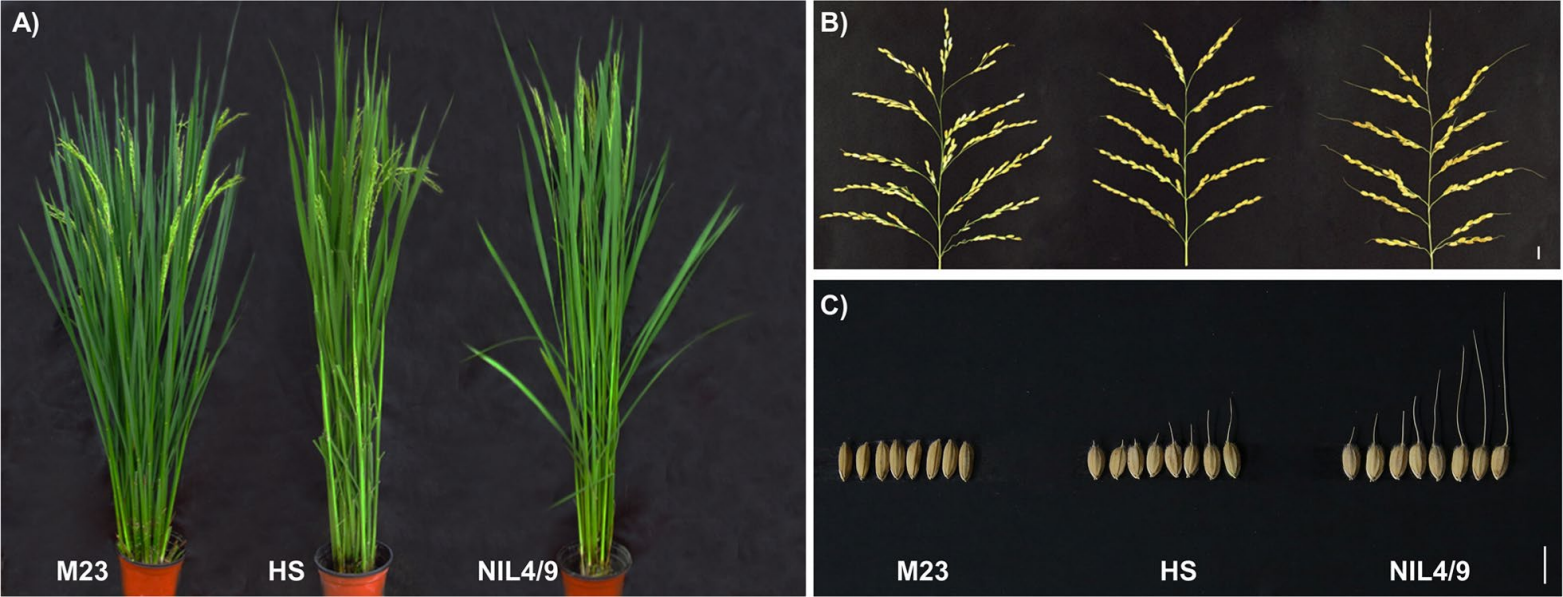
Phenotypic characterization of parental lines. **A** Plant morphology, **B** panicle type, and **C** awn length of Milyang23 (M23), Hwaseong (HS), and NIL4/9. Scale bar = 1 cm

**Fig. 2:**
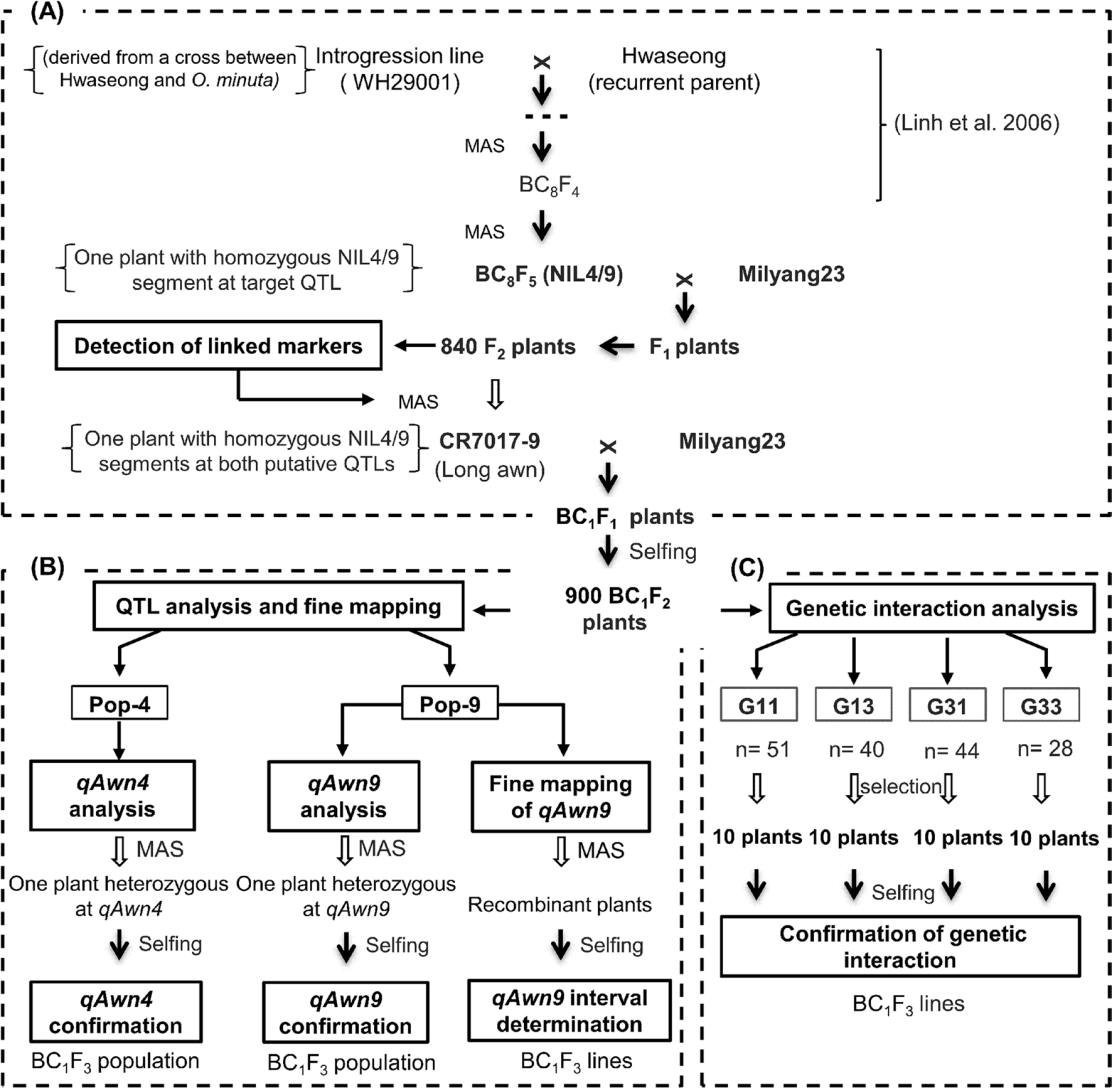
Schematic of the experimental strategy used to perform QTL mapping. **A** Development of the F_2_ population from a cross of NIL4/9 and Milyang23. An F_2_ plant was chosen and crossed to Milyang23 to produce BC_1_F_1_ plants. **B** Flow diagram of population development to map two QTLs, *qAwn4* and *qAwn9,* and to fine map *qAwn9*. Each BC_1_F_2_ plant was chosen to map *qAwn4* and *qAwn9*. Recombinant plants in the *qAwn9* region were selected and advanced to BC_1_F_3_ lines for fine mapping of *qAwn9*. **C** Four groups with different gene combinations of *qAwn4* and *qAwn9* were selected to analyze the interaction of two QTLs

A near-isogenic line, NIL4/9, harboring an *O. minuta* (IRGC No. 101141) segment on chromosome 9 was selected from the BC_8_F_4_ population derived from a cross between *O. minuta* and Hwaseongbyeo (hereinafter Hwaseong) in a previous study (Linh et al. 2006). To identify the QTLs influencing awn development in NIL4/9 and *japonica* cultivar Hwaseong, a cross was made between the Korean *indica* cultivar Milyang23 (awnless phenotype) and NIL4/9 (long awn) (Fig. 1). The F_2_ population (840 plants) derived from F_1_ plants was used to detect additional QTLs other than *qAwn9*. To detect QTLs, two contrasting phenotypic groups, each with 11 long-awned and 11 awnless Milyang23 homozygous F_2_ plants at *qAwn9*, were screened, and one additional major QTL, *qAwn4*, was observed. Based on the genotypes at two major QTLs, *qAwn4* and *qAwn9*, one long-awned F_2_ plant, CR7017-9, with homozygous NIL4/9 alleles at *qAwn4* and *qAwn9,* was selected and backcrossed with Milyang23 (Fig. 2A). By selfing the BC_1_F_1_ plants, 900 BC_1_F_2_ individuals were generated for QTL confirmation, fine mapping, and genetic interaction analysis.

### Field Experiments and Phenotyping

Germinated seeds were first sown in trays, placed under greenhouse conditions and then moved outdoors for one week before transplanting. At 28 days after sowing, seedlings (one plant per hill) were transplanted at a distance of 15 cm between plants within a row and 30 cm between rows. All plant materials (crossing and natural populations) were grown in a bird-net-equipped field at Chungnam National University, Daejeon, South Korea. Plants with awns longer than 1 mm were used to measure the length with a millimeter ruler, while the others were considered awnless phenotypes. Three main panicles were collected from each plant for phenotyping. The average awn length of the apical spikelets on each primary branch was used to represent the awn length of the whole panicle.

### Analysis of QTL and Interaction Analysis for Seed Awning

QTL analysis for seed awning was conducted using a BC_1_F_2_ population to confirm the linkage between the candidate markers and awn QTLs. To minimize the influence of putative QTLs on each other, two subgroups, Pop-4 and Pop-9, were selected based on the genotypes of 900 BC_1_F_2_ plants at two QTLs, *qAwn4* and *qAwn9*, respectively (Fig. 2B). In Pop-4, all plants segregated at *qAwn4* and were Milyang23 homozygous at *qAwn9*. All the plants in Pop-9 segregated at *qAwn9*, and were Milyang23 homozygous at *qAwn4*. A putative QTL was estimated using single marker analysis or interval marker analysis based on one-way ANOVA followed by Tukey’s test. Additionally, a single plant heterozygous for the putative QTL was selected and selfed in each subgroup. QTL analysis for seed awning was repeated using BC_1_F_3_ populations to confirm the QTL detection.

To determine the interaction between *qAwn4* and q*Awn9*, four homozygous genotypes (G11, G13, G31, and G33) from the BC_1_F_2_ population were selected using three markers (RM252 for *qAwn4* and RM201 and RM24751 for *qAwn9*). G11 was homozygous for Milyang23 at *qAwn4* and *qAwn9*, whereas G13 was homozygous for Milyang23 at *qAwn4* and *O. minuta* at *qAwn9*. In addition, G31 was homozygous for Hwaseong at *qAwn4* and Milyang23 at *qAwn9*, and G33 was homozygous for Hwaseong at *qAwn4* and *O. minuta* at *qAwn9.* For each genotype G11, G13, G31, and G33, ten BC_1_F_2_ plants were selected to account for possible background effects*.* Moreover, the awn length of ten BC_1_F_3_ lines (21 plants per line) in each group was evaluated to confirm the genetic interactions (Fig. 2C).

### DNA Extraction and Genotype Analysis

Total DNA was extracted from fresh leaves using the CTAB method described by (Murray and Thompson 1980). The polymerase chain reactions (PCR) were carried out in a 15-μL reaction volume containing 2-μL of diluted genomic DNA, 0.15-μL of 5.0 U/μL Taq DNA polymerase (Elpis), 1.5-μLl of dNTP Mix (2.5 uM each), 1.5-μL of Primers mix (10 pmol/μL), 1.5-μL of 10X PCR buffer, and 8.35 μL of ddH_2_O. The amplified products were electrophoresed on a 3% high-resolution MetaPhor agarose gel (Lonzan, Rockland, ME, USA) or a 4% polyacrylamide denaturing gel, depending on the band separation of the markers. The same procedure was used for genotyping all materials in this study. Markers used to analyze the two awn QTLs, and fine-map *qAwn9* are described (Additional file 2: Table S1).

### Genome-Wide Association Study (GWAS)

The *qAwn9* was firstly detected by using a population derived from interspecific cross between *O. sativa* and wild rice *O. minuta*. However, we were interested in knowing whether or not the association between *qAwn9* and awn length variation also existed in natural population of cultivated rice *O. sativa*. We performed GWAS using the sequence information of 137 accessions in the KRICE_CORE set listed in Additional file 2: Table S2 and the method described in a previous study (Kim et al. 2016). Association analysis for awn development was performed using the compressed mixed linear model (MLM) method using GAPIT, where the PCA and relative kinship matrices were defined as fixed and random effects. Significant single nucleotide polymorphisms (SNPs) were identified at a significance threshold of -log_10_(*p*) > 6.0.

### High-Throughput Whole-Genome Sequencing

High-throughput whole-genome sequencing was performed on three parental lines, NIL4/9, Hwaseong, and Milyang23. Whole-genome sequencing was performed by Macrogen Inc. (Beotkkot-ro, Geumcheon-gu, Seoul, South Korea). Test-qualified genomic DNA samples were used to construct shotgun DNA libraries using the TruSeq Nano DNA Kit (San Diego, California 92,122, USA) and were sequenced using HiSeq X Ten. The project result is summarized in Additional file 2: Table S3.

### Analysis of Candidate Genes

Potential candidate genes were shortlisted based on SNPs and insertions/deletions (InDels) in the promoter and exon regions between parental lines. Haplotype analysis was performed on the most likely candidate genes using all SNPs within the gene coding sequence region, in which synonymous SNPs were ignored. Phenotypic differences between the haplotypes were estimated in the KRICE_CORE set based on the *t*-test (n > 5 rice accessions). Additionally, the sequence information of 36 *Oryza rufipogon* accessions was downloaded from the TASUKE database (Additional file 2: Table S4, https://rapdb.dna.affrc.go.jp).

## Results

### Genetic Background of NIL4/9

**Fig. 3:**
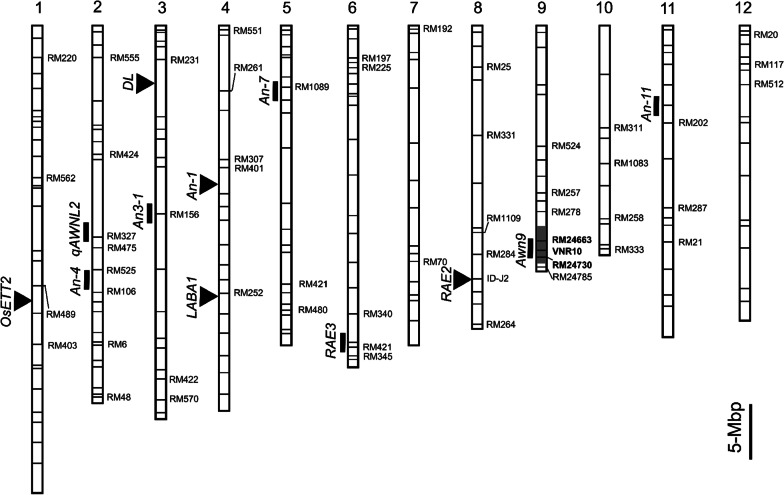
Distribution of 212 markers and known genes/QTLs for awn development on 12 chromosomes. Polymorphic markers between Milyang23 and NIL4/9 are displayed with names, while bold markers are polymorphic between Hwaseong and NIL4/9. Markers without names indicate monomorphic/ambiguous markers. Cloned genes and QTLs for awn development detected by previous studies are indicated by black triangles and black boxes, respectively, on the left side of chromosomes. Gray box indicates the homozygous *O. minuta* segment detected by RM24663, VNR10, and RM24730. The bar indicates 5-Mbp

To detect *O. minuta* segments in NIL4/9, 212 SSR markers distributed across the 12 rice chromosomes were screened between Hwaseong and NIL4/9. Consequently, with several continuous backcross generations combining marker-assisted selection, one *O. minuta* segment was detected by three markers, RM24663, RM24730, and VNR10, on chromosome 9, which overlapped with the region in our previous study (Fig. 3). Notably, two markers, RM24663 and VNR10, produced polymorphic bands in the three parental lines. Furthermore, when the F_2_ population was screened with these two markers, all F_2_ plants carried the *O. minuta* segment and/or Milyang23, whereas no Hwaseong segment-carrying plants were detected (Additional file 1: Fig. S1). These results further confirmed the presence of *O. minuta* segment in the NIL4/9 background.

### First Signature of Multiple QTLs Associated with Awn Length

NIL4/9 was generated by repeated backcrossing with selection for introgression of an *O. minuta* segment into the Hwaseong background. On average, the awn length of NIL4/9 was 13.30 ± 1.46 mm, and that of Hwaseong was 5.50 ± 0.99 mm. Compared to Hwaseong, NIL4/9 showed significantly longer awns, suggesting that NIL4/9 carried additional genes/QTLs associated with seed awning; one was *qAwn9*, detected in our previous study, and the other genes were possibly segregated in the Hwaseong background. This hypothesis is consistent with the phenotypic data of the F_2_ population (derived from a cross between Milyang23 and NIL4/9) that *qAwn9* alone cannot explain the variation in awn phenotypes.

To detect additional QTLs for awn development, 54 polymorphic markers between NIL4/9 and Milyang23 were selected out of 212 markers (Fig. 3). Two contrasting phenotypic groups, each with 11 long-awned and 11 awnless Milyang23 homozygous F_2_ plants at *qAwn9*, were screened using 54 markers. Genotype analysis showed that at RM252 on chromosome 4, eight long-awned plants and all 11 awnless plants were homozygous for the Hwaseong and Milyang23 alleles, respectively, and three long-awned plants were heterozygous (χ^2^ = 22, *p* < 0.001), implying that RM252 is likely to be linked to the causal QTL controlling awn development in this population (Additional file 1: Fig. S2). However, the other markers failed to show consistent polymorphic patterns between the two groups (ID-J, χ^2^ = 1.81, *p* = 0.40; RM525, χ^2^ = 0.40; *p* = 0.82), suggesting that these markers were not linked to the additional QTL. Hence, RM252 was used as a marker to analyze additional QTL. Considering that this procedure only allows for approximate mapping of the QTL, further mapping analysis using a backcross population was conducted.

### QTL Analysis for Seed Awning

QTL analysis for seed awning was conducted using BC_1_F_2_ individuals to confirm the two respective QTLs. One was linked to RM252 on chromosome 4 (*qAwn4*), and the other was located on chromosome 9 showing linkage to two markers, RM201 and RM24751 (*qAwn9*) (Table 1 and Additional file 1: Fig. S3). *qAwn4* accounted for 19.1% of total phenotypic variation. *qAwn9* was further confirmed in this study and accounted for 14.6% of phenotypic variation. QTL analysis was conducted using BC_1_F_3_ progenies to confirm the accuracy of QTL detection. Both QTLs were repeatedly detected in two consecutive years, and *qAwn4* and *qAwn9* accounted for 21.6% and 20.0% of phenotypic variation, respectively. These results confirmed that both QTLs had a major effect (R^2^ > 14%) and that awn length is a complex quantitative trait controlled by multiple genes and the environment. Additionally, the awn length of the heterozygotes was intermediate between that of the two homozygotes, indicating that Hwaseong and *O. minuta* alleles act additively at *qAwn4* and *qAwn9*, respectively.

**Table 1:** QTL analysis for awn development in BC_1_F_2_ and BC_1_F_3_ populations

Generation	n^a^	QTL	Chr	Marker	*p*-Value	R^2^ (%)^b^	Known gene
BC_1_F_2_	900	*qAwn4*	4	RM252	< 0.001	19.1	*LABA1*
		*qAwn9*	9	RM201-RM24751	< 0.001	14.6	
		*qAwn4/qAwn9*			0.028		
		Total^c^				53.6	
BC_1_F_3_	240	*qAwn4*	4	RM252	< 0.001	21.6	*LABA1*
BC_1_F_3_	277	*qAwn9*	9	RM201-RM24751	< 0.001	20.0	

^a^n: Population size

^b^R^2^: Phenotypic variance was determined using one-way ANOVA

^c^Total phenotypic variance was determined using regression analysis

Interestingly, *qAwn4* overlapped with the well-known gene *LABA1*. Therefore, sequencing analyses of the parental lines were conducted to determine whether *LABA1* in the QTL region was the causal gene for *qAwn4* in this population. Notably, the previously known functional variation of *LABA1,* a 1-bp deletion at + 69-bp in the first exon, was discovered (Additional file 1: Fig. S4, (Hua et al. 2015)). Therefore, *qAwn4* is designated as *LABA1* hereinafter. Furthermore, based on the genetic background of NIL4/9, we determined that the *O. minuta qAwn9* allele and the Hwaseong *LABA1* allele contributed to an increase in awn length, whereas Milyang23 had nonfunctional alleles at both loci.

### Interaction Between *LABA1 *and q*Awn9*

**Fig. 4:**
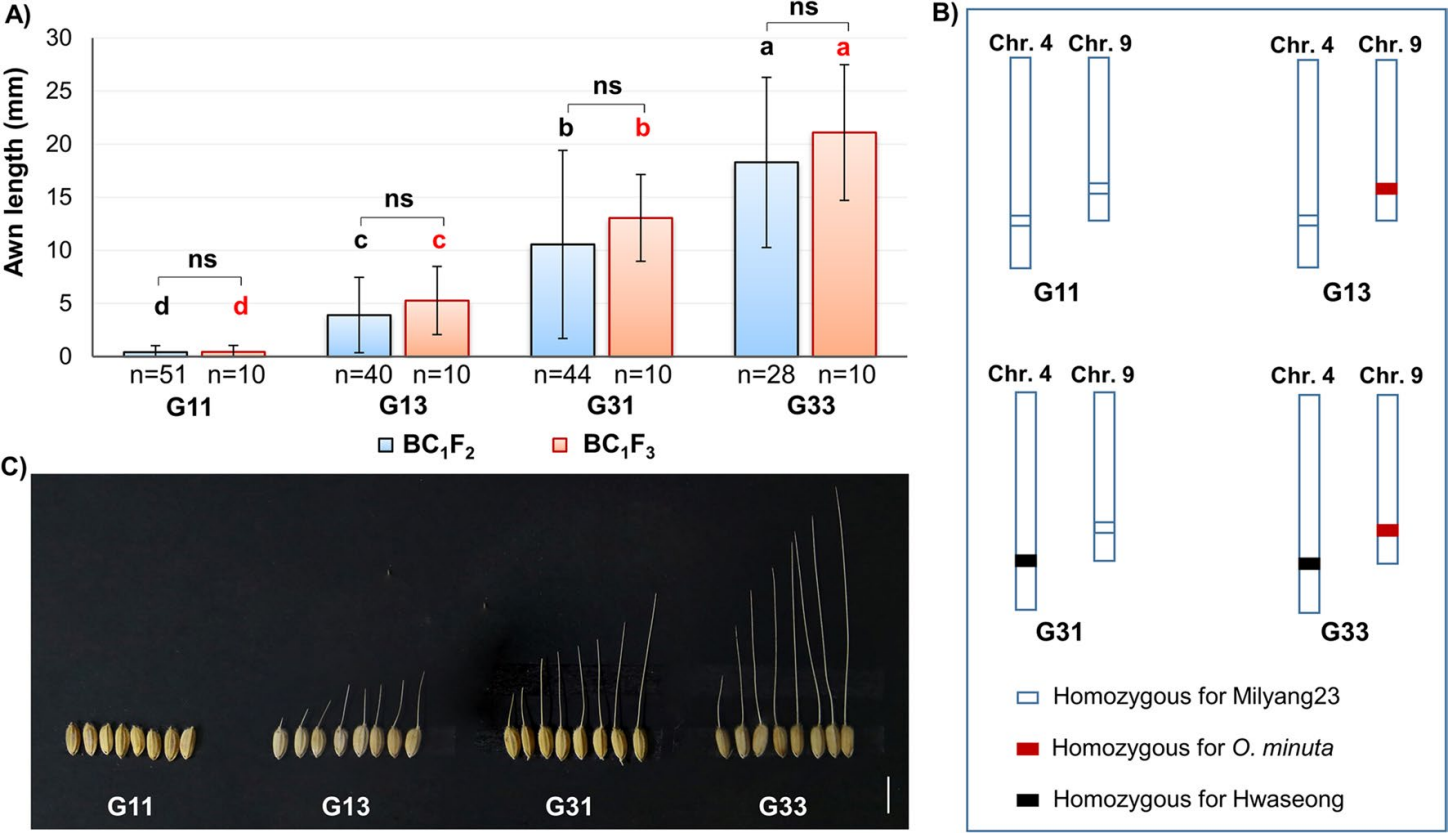
Comparison of awn length of the four genotype groups in BC_1_F_2_ and BC_1_F_3_ generations. **A** Means labeled with different letters are significantly different among genotypes (Tukey’s test, *p* < 0.05). The error bar indicates the standard deviation. **B** Graphical genotypes of four genotype groups. **C** The photograph shows variations in awn length of four genotype groups. Scale bar = 1 cm

In this study, two QTLs were detected for awn length, and their interaction with seed awning was further examined. Analysis indicated a significant interaction between *LABA1* and *qAwn9* (*p* = 0.028) (Table 1). To characterize the interaction between *LABA1* and *qAwn9*, four different combinations of homozygous genotypes (G11, G13, G31, and G33) were compared in terms of awn length. The average awn lengths of the four genotypes were significantly different (Fig. 4). G11 plants (with Milyang23 homozygous alleles at both loci) had no or tip awn, whereas awn formation and elongation were observed in plants with functional alleles at one of the two loci. The awns of the G13 and G31 plants (having the functional allele at *qAwn9* or *LABA1*) were significantly longer than those of the G11 plants. This result indicates that both loci can independently induce awns. The longest awns were observed in G33 plants, with functional alleles at both loci. The interaction was also observed for awn length when ten BC_1_F_3_ lines (21 plants per line) of each group were evaluated. The awn length in each group showed a wide range of variation, especially in BC_1_F_2,_ probably because of the difference in the genetic background of the individual BC_1_F_2_ plants used to measure awn length. Compared to the BC_1_F_2_ plants, the variation in awn length was reduced in the BC_1_F_3_ line. Moreover, the possibility that some minor QTLs segregated in the population could not be ruled out because only the large-effect *qAwn4* was detected using two extreme phenotype groups in the F_2_ population derived from a cross between Milyang23 and NIL4/9. Altogether, *qAwn9* and *LABA1* behaved additively during awn development and showed a stable and major effect in Milyang23 (*indica*) and Hwaseong (*japonica*) backgrounds.

### Identification of Genomic Regions Associated with Awn Length in the Natural Population

GWAS analysis identified 16 genomic regions (designated as QTLs) tagged with 48 significant SNPs. These SNPs were localized on chromosomes 1, 2, 3, 4, 7, 8, 9, 11, and 12, which explained 32–43% of the phenotypic variation of the trait (Additional file 1: Fig. S5 and Additional file 2: Table S5). Among them, several significant associations overlapped or were closely linked to known genes or previously detected QTLs. *qAwn4* and *qAwn8.2* overlapped with the two major genes, *An-1* and *RAE2*, respectively (Bessho-Uehara et al. 2016; Luo et al. 2013). *qAwn2.2* is located close to *qAWNL2* and *An-4* (Amarasinghe et al. 2020; Qin et al. 2021). *qAwn11.1* overlapped with an awn QTL on chromosome 11 (Bessho-Uehara et al., 2021). These co-locations of the known genes/QTLs detected in previous studies could provide proof of the accuracy of our analysis. Notably, this study mapped the significant associations at position 20.6 Mbp on chromosome 9 overlapped with *qAwn9*. This result suggests further investigation for the fine mapping of *qAwn9*.

### Fine Mapping of *qAwn9*

**Fig. 5:**
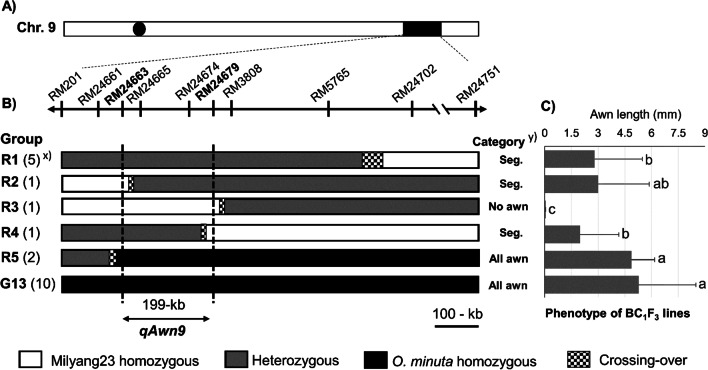
Fine mapping of *qAwn9*. **A** Chromosomal location of *qAwn9*. **B** Genotypes of five groups of BC_1_F_2_ recombinant plants and homozygous lines G13 as the control. Black, white, and gray bars denote *O. minuta,* Milyang23, and heterozygous segments, respectively. **C** Phenotypes of BC_1_F_3_ lines. Means labeled with different letters are significantly different among lines (Tukey’s test at *p* < 0.05). ^x)^ Numbers in parenthesis indicate the number of plants. ^Y)^ “Seg.” means segregation for awning that was determined by a *t*-test between two homozygous plants. Progeny tests of recombinants delimited *qAwn9* to an approximately 199-kb region flanked by markers RM24663 and RM24679

For the fine mapping of *qAwn9*, 50 BC_1_F_2_ plants with the Milyang23 allele at *LABA1* and recombination events between RM201 and RM24751 on chromosome 9 were selected. Fifty BC_1_F_2_ recombinant plants were examined using additional markers to better determine crossover breakpoints. The plants in each recombinant group were then selfed to produce BC_1_F_3_ lines that were used for phenotype measurements (Fig. 5 and Additional file 2: Table S6). Based on the combination of genotypes and phenotypes of five groups of recombinant plants (R1 to R5) and the control (G13), the *qAwn9* location was mapped within an interval of 199-kb between two SSR markers, RM24663 and RM24679. Within the 199 kb region, 27 genes were annotated based on the RAP-DB database (https://rapdb.dna.affrc.go.jp). Among them, five genes were predicted as hypothetical genes or encoded proteins of unknown function, whereas the other 22 genes showed functional annotations (Additional file 2: Table S7).

### DNA Sequence Variants of Candidate Genes Underlying *qAwn9*

To identify candidate genes for *qAwn9*, the genomic region of *qAwn9* spanning 199-kb was directly analyzed by whole-genome sequencing of parental lines. We focused on SNPs and InDels in the promoter and exon regions that lead to non-synonymous mutations. Out of 27 genes, five hypothetical genes or encoded proteins of unknown function were omitted. The sequence variants of the remaining 22 genes were examined. Potential candidate genes were shortlisted based on sequence variants between parental lines. One gene, Os09g0520400, displayed three missense variations in exon regions; four genes, Os09g0521400, Os09g0521900, Os09g0524800, and Os09g0525400, showed sequence variants in their promoters; and remaining 17 genes showed no missense variation or variation in their promoters (Additional file 1: Fig. S6 and Additional file 2: Table S8).

### Haplotype Analysis of the Candidate Genes

**Fig. 6:**
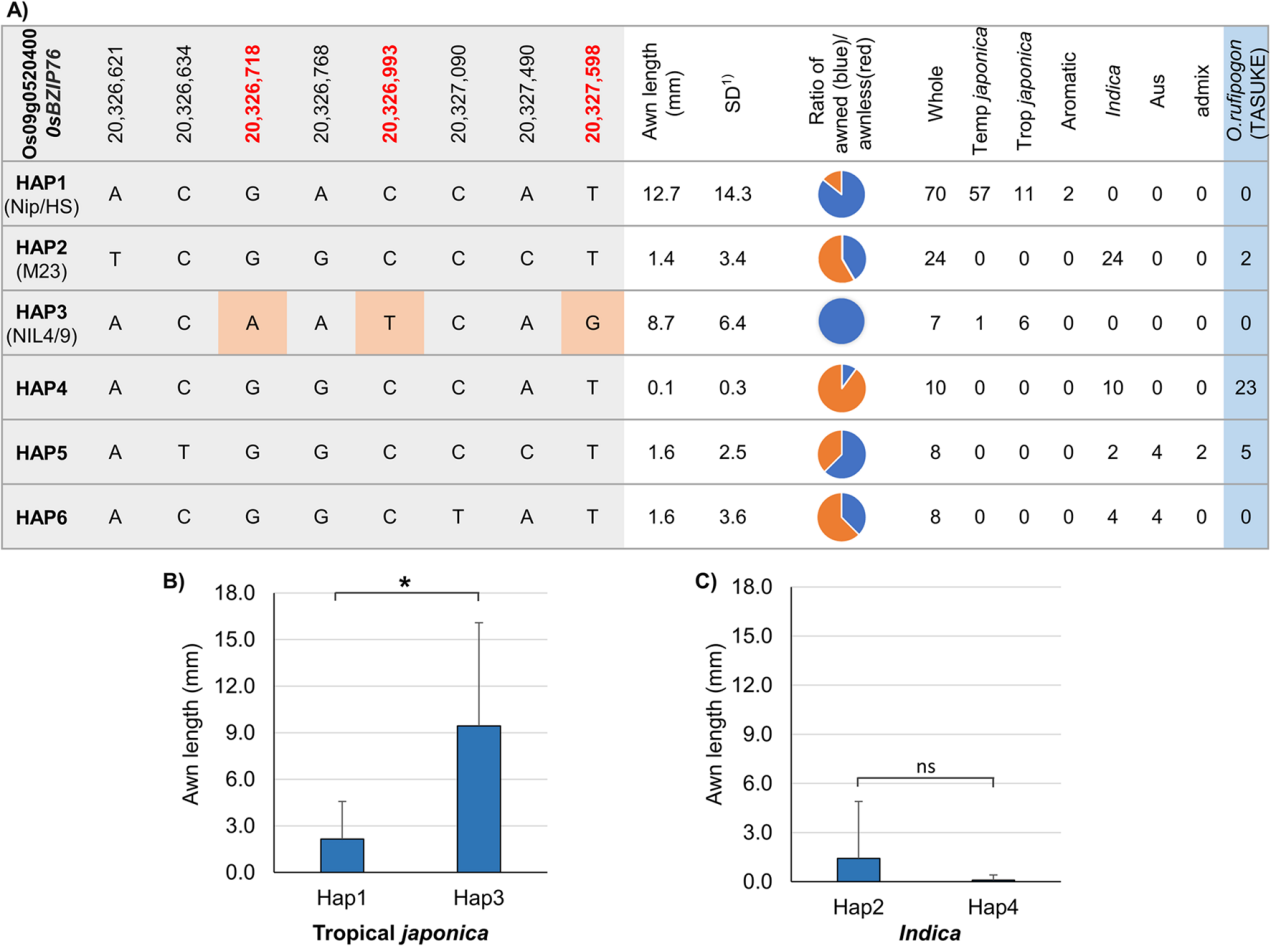
Variations in Os*bZIP76* were significantly associated with awn development. Haplotype analysis of Os*bZIP76* for awn length (**A**). Comparison of awn lengths in different haplotypes in tropical *japonica* (**B**) or *indica* (**C**). * and *ns* significant at *p* < 0.05 and not significant, respectively (*t*-test). ^1)^*SD* standard deviation

Haplotype analysis was conducted to examine the possible functional variation of *qAwn9* since *qAwn9* was detected in GWAS using the KRICE_CORE set. Based on the functional annotations and sequence variations, we considered *OsbZIP76* (Os09g0520400*,* a transcription factor gene) as the strongest candidate gene for haplotype analysis. Of the 137 accessions in KRICE_CORE set, 127 were classified into six haplotypes of *OsbZIP76* based on eight SNPs in the coding region shared by at least seven accessions (Fig. 6A and Additional file 2: Table S2). The remaining 10 accessions could not be classified owing to heterozygous and/or missing data. Haplotype 1 (Hap1) was the most common and was present in 70 (55.1%; n = 127) accessions. It was also the most frequent haplotype within temperate *japonica* (98.3%; *n* = 58), and Hwaseong belonged to the Hap1. Haplotype 2 (Hap2) included 24 (18.9%; n = 127) accessions, and the predominant haplotype within *indica* (60%; *n* = 40) and Milyang23 belonged to Hap2. Haplotypes 4, 5, and 6 were predominantly carried by the *indica* or *aus* accessions. These haplotypes were relatively minor (6.3–7.9%; n = 127). Notably, Haplotype 3 (Hap3) was rare in the KRICE_CORE set (5.5%), absent in *indica*, *aus*, and *aromatic*, and found in one temperate *japonica* accession, but it was the common haplotype within tropical *japonica*, present in 35.3% (n = 17) of accessions, and NIL4/9 belonged to Hap3. Compared to the awn development across the six haplotypes, Hap1 and Hap3 had significantly longer awn of 12.7 and 8.7 mm than Hap2, Hap4, Hap5, and Hap6 with the mean awn length of 1.4, 0.1, 1.6, and 1.6 mm (Fig. 6A). We found that 53 (75.7%) of the 70 rice accessions in Hap1 contained the functional *LABA1* allele (based on the presence or absence of the functional 1-bp deletion at the + 69 bp site; Chr. 4:25,959,586), which partly explains the long awn of the accessions in Hap1. In contrast, in Hap3, only one temperate *japonica* accession had a functional allele of *LABA1,* whereas the other six tropical *japonica* accessions had the nonfunctional *laba1* allele, suggesting that the awn development of tropical *japonica* accessions might not be controlled by *LABA1.* All seven accessions of Hap3 showed awns. Therefore, we hypothesized that Hap3 carries a functional mutation that is associated with awn development. Given that the population structure (corresponding to subspecies) might lead to a false positive association between the awn length and haplotypes, a further comparison of the awn length between haplotypes within each subspecies was performed. Only tropical *japonica* and *indica* had sufficient accessions to at least two haplotypes for comparison. Consequently, Hap3 had significantly longer (*t*-test, *p* = 0.02) awns of 9.4 mm than Hap1, which had a mean awn length of 2.1 mm (Fig. 6B). However, no significant difference in awn length was observed between Hap2 and Hap4 in *indica* (Fig. 6C). This provided further evidence that Hap3 might have functional variation in awn development.

Additionally, we examined the sequence information of 36 *O. rufipogon* accessions from the TASUKE database based on the eight non-synonymous SNPs in *OsbZIP76*. Surprisingly, none of the *O. rufipogon*s belonged to Hap3, although they were mainly classified as Hap4 (Fig. 6A and Additional file 2: Table S4). Therefore, we conclude that the Hap3 allele might be tropical *japonica*-specific, different from *O. rufipogon*, and has three non-synonymous SNPs in *OsbZIP76*.

To further investigate the extent of sequence variation of *OsbZIP76* across *O. sativa* subpopulations, we built a network of haplotypes using the tool ‘haplotype network analysis’ implemented in RiceVarMap (ricevarmap.ncpgr.cn). A consistent frequency result for the six haplotypes was obtained (Additional file 1: Fig. S7A). Hap1 and Hap2 were still the most common haplotypes in the dataset and were the predominant haplotypes in *japonica* and *indica*, respectively, whereas Hap3 was the predominant haplotype in tropical *japonica*. For further detail, we examined the SNP-Seek database containing re-sequencing information for a collection of 3 K *O. sativa* accessions (http://snp-seek.irri.org). Notably, Hap3 differs from the other five haplotypes in three missense SNPs (Chr. 9:20,326,718, 20,326,993, and 20,327,598) that we previously detected in NIL4/9, Milyang23, and Hwaseong. Interestingly, among the SNPs detected in the 3 K population, these three missense mutations were predominantly observed in tropical *japonica,* whereas they were rare in other subspecies (Additional file 1: Fig. S7B). Based on these observations, we speculated that Hap3 is a tropical *japonica-*specific haplotype, and Hap3 could cause phenotypic variations.

## Discussion

The genetic diversity of parental lines is essential to enhance the success rate of detecting QTL/genes for complex traits. In this study, a population was developed from a cross between an *indica* cultivar Milyang23 with no awn and NIL4/9 with long awns that allowed us to (1) understand the effect of *qAwn9* in different genetic backgrounds, (2) simultaneously detect and analyze the other QTLs for awn length and their genetic interactions in the same cross population, and (3) analyze QTLs with higher precision and resolution because of the reduced effect of unknown QTLs on awn length.

QTL mapping is further complicated by the requirement to resolve the effect of one genetic locus from the noise created by segregation at other genetic loci that also influence the trait of interest. To minimize the influence of putative QTLs on each other, two subgroups were selected based on the genotypes and phenotypes of 900 BC_1_F_2_ plants at the two putative QTLs, *qAwn4* and *qAwn9*. For *qAwn4* analysis, a single plant heterozygous for *qAwn4* and Milyang23 homozygous for *qAwn9* was selected. Moreover, a single plant heterozygous for *qAwn9* and Milyang23 homozygous for *qAwn4* was selected to analyze *qAwn9*. This strategy allowed us to analyze QTLs for awn length as a single Mendelian factor, which increased the statistical power and accuracy of the QTL linkage relationship. Consequently, two QTLs for awn were repeatedly detected in two consecutive generations, BC_1_F_2_ and BC_1_F_3_, indicating the accuracy of QTL mapping. Moreover, *LABA1* was determined to be the causal gene for *qAwn4.* The Hwaseong allele contributed to awn development at this locus which is consistent with the fact that many temperate *japonica* accessions have an awned phenotype and harbor the functional allele of *LABA1* (Hua et al. 2015). QTL analysis also detected a major QTL for awn length on chromosome 9, where the wild rice *O. minuta* allele increased awn length. However, the two QTLs identified in this study are only the minimum estimates of the actual number of loci influencing awn development. The parental lines used in our study may differ from other minor loci that affect awn development. For instance, some plants with Milyang23 homozygous alleles at both loci, which were expected to have no awn, showed a short-awned phenotype. This phenomenon suggested the presence of other loci with minor effects.

### Interaction between *LABA1 *and *qAwn9*

In addition to understanding the function of a single gene or QTL, the characterization of the genetic interactions among them is also important. In this study, an interaction between *LABA1* and *qAwn9*, which is involved in awn development, was detected in the BC_1_F_2_ and BC_1_F_3_ populations (*p* = 0.028, Table 1). However, the confidence in their detection of interactions is relatively low because of the use of the BC_1_F_2_ segregating population, which segregates parental chromosomal segments simultaneously at multiple loci. Additionally, environmental conditions are considered as factors influencing awn development. Nevertheless, the consistent results for both generations demonstrated the power of the experiment, and this population was eligible for the analysis. *qAwn9* and *LABA1* behaved additively during awn development and showed a stable and major effect in Milyang23 (*indica*) and Hwaseong (*japonica*) backgrounds. These results are consistent with those of previous studies. For example, three major genes for seed awning, *An-1*, *LABA1,* and *RAE2*, were detected to have additive effects on awn development in the genetic background of cultivated rice (Ikemoto et al. 2017; Jin et al. 2016). Similarly, a minor QTL, *qAWNL2*, had an additive effect with *An-1* and *LABA1* (Amarasinghe et al. 2020). The same type of interaction has also been demonstrated in a recent study between *An-4* and other genes involved in awn development, such as *An-1*, *LABA1*, and *RAE2* (Qin et al. 2021). These studies indicate that the effects of such interactions are common for awn development in rice.

### Fine Mapping of *qAwn9*

*qAwn9* represents a major and stable locus for awn development that is expressed in Hwaseong and Milyang23 backgrounds. To fine-map *qAwn9* as a single Mendelian factor, we selected BC_1_F_2_ plants with Milyang23 homozygous alleles at *LABA1* and recombination break points in the *qAwn9* region. In addition to considering the awn length of each BC_1_F_2_ plant, segregation analysis of the awn length of the two homozygous genotypes in the BC_1_F_3_ progenies of informative F_2_ recombinant plants was also conducted. Segregation analysis of the BC_1_F_3_ progenies was valuable for delimiting the location of the causal QTL, considering the effect of the environment or an unknown gene on the awn phenotype in the population. Theoretically, approximately half of the progeny of BC_1_F_2_ recombinant plants are heterozygous at a given locus, which may impede the precise evaluation of their phenotypes in mapping. Combining the awn length and genotypes of two homozygous BC_1_F_3_ progeny from each recombinant plant allowed us to infer whether the target locus was segregated. Based on segregation analysis and substitution mapping, *qAwn9* was mapped to a 199-kb physical region between markers RM24663 and RM24679.

### Candidate Gene for Awn Development

Based on the list of genes annotated in the *qAwn9* region, five hypothetical genes or encoded proteins of unknown function were omitted. We compared the sequences of 22 remaining genes between parental lines and found four genes, Os09g0521400, Os09g0521900, Os09g0524800, and Os09g0525400, had SNP or deletion variations in the promoter region, whereas the transcription factor gene, Os09g0520400 *(OsbZIP76)*, had three missense variations in the coding sequence. Many studies have characterized awn genes in rice and barley, most of which encode transcription factors or enzymes. As mentioned above, *LABA1* encodes a cytokinin synthesis enzyme, whereas *RAE2* is predicted to encode a small secretory signal peptide belonging to the EPIDERMAL PATTERNING FACTOR-LIKE family (Hua et al. 2015; Jin et al. 2016). Notably, three genes, *An-1*, *TOB1*, and *DL*, are transcription factors. *An-1* encodes a bHLH transcription factor (Luo et al. 2013), whereas *TOB1* and *DL* encode the YABBY transcription factors (Toriba and Hirano 2014; Tanaka et al. 2012). In barley, three major genes have been identified at the molecular level, all of which encode transcription factors: six-rowed spike 1 (*vrs1)*, hooded lemma 1 (*Kap1)*, and short awn 2 (*lks2)* (Huang et al. 2021). Of these, two genes, *vrs1 and lks2,* harbor missense mutations that are functional variations in awn development. Transcription factors are proteins that typically regulate the expression of a group of genes involved in a pathway or developmental process such as awn initiation and elongation. Os*bZIP76,* encoding a transcription factor, also revealed three missense mutations in variant calling between the parental lines. Therefore, it is unsurprising that candidate gene Os*bZIP76* is the causal gene underlying *qAwn9*. Moreover, a further comparison of awn length between the haplotypes of Os*bZIP76* within the tropical *japonica* subpopulation showed that Hap3 had significantly longer awns than Hap1. This finding provides further evidence that Os*bZIP76* may be a target gene. However, additional experiments, such as gene transformation, are required to confirm the function of Os*bZIP76* in awn development*.*

### *O. minuta*: A Valuable Resource to Identify a New Awn Gene

Previous studies have used recombinant inbred lines and advanced backcross populations developed from crosses between an *indica*/*japonica* of *O. sativa* and a strain of *O. rufipogon* to detect QTLs and identify their underlying genes (Shim et al. 2020; Ngu et al. 2014). Three major genes, *An-2, RAE2*, and *An-1*, have been cloned using chromosome segment substitution lines derived from interspecific cross between *O. sativa* and *O. rufipogon* (Luo et al. 2013; Gu et al. 2015; Ikemoto et al. 2017). Some minor QTLs, *qAWNL2* and *An-4*, have also been detected using populations derived from interspecific crosses (Amarasinghe et al. 2020; Qin et al. 2021). Recently, a study explored the loci responsible for awn development in rice through a comparative analysis of all AA genome species (Bessho-Uehara et al. 2021). Most known genes/QTLs for awn development were also detected, and two loci on chromosomes 7 and 11 were newly identified in that study. However, *qAwn9* was not identified in previous studies. The wild rice *O. minuta* (2n = 48, BBCC) is an allotetraploid genome species in the *Oryza officinalis* complex and has the second largest genome size among *Oryza* species, at 1124 Mbp (Elgamal and Elshenawy 2018). It has accumulated abundant genetic diversity and harbors a long awn. Thus, it can be used as a donor for developing mapping population and will probably lead to the discovery of novel genes involved in awn development in rice. The cross combinations between Milyang23/Hwaseong and *O. minuta* could explain why *qAwn9* was detected in our studies but was not mapped in the above studies. Indeed, extensive genetic research based on haplotype analysis in this study indicated that Hap3 of *OsbZIP76* in the *qAwn9* region is rare in aus, *indica*, temperate *japonica*, and *O. rufipogon* strains and was exclusively observed in tropical *japonica* and *O. minuta*. Therefore, the special long-awned line NIL4/9 is very valuable and provides a material base for the present study. However, we cannot currently provide evidence on how the unique alleles of Hap3 were produced, although it is possible that natural mutations in the *qAwn9* genomic region within tropical *japonica* adapted to the environmental conditions in a unique domestication history or the accessions containing clustered sequences, including Hap3, may still retain the original alleles of their wild progenitors. A similar result was found when other genes surrounding *OsbZIP76* were screened. Two genes, Os09g0520100 and Os09g0521300, also contained unique alleles in tropical *japonica,* indicating the presence of a tropical *japonica-*specific clustered sequence in this region (Additional file 1: Fig. S8). In addition, the unique alleles of Os09g0520100 at position 20,315,260 were the same as those detected in *O. minuta.* It is unclear whether there is any close relationship between tropical *japonica* and *O. minuta* because of the same exclusive alleles detected in this study and the same local origin in Indonesia, the Philippines, and the southern part of Indochina as reported in a previous study (Konishi et al. 2008; Elgamal and Elshenawy 2018) or it possibly occurred by crossing between tropical *japonica* and *O. minuta.* Much remains to be learned to provide further insight into rice domestication.

## Conclusion

In the present study, QTL analysis revealed that two loci, *LABA1* and *qAwn9*, were mainly responsible for awn development in NIL4/9. The combination of *LABA1* and *qAwn9* showed an additive effect, and both loci could independently induce awn. Therefore, to control awnlessness via breeding programs, the accumulation of natural variations at all awning loci should be considered. The *qAwn9* was fine-mapped in our study using an interspecific cross between *O. minuta* and cultivated rice, supporting the importance of wild rice as a resource that is valuable for rice breeding and provides a potential opportunity for identifying novel genes. *qAwn9* is an important molecular target for understanding the genetic control of awn development in rice. This study provides novel insights into the genetic architecture of awn development and establishes the basis for candidate gene analysis to lay a foundation for further cloning of the awn gene underlying *qAwn9*.

## Supplementary Information

**Additional file 1**: **Fig S1** Confirmation of the O. minuta segment in the NIL4/9 background. The O. minuta and Milyang23 alleles were segregated at the RM24663 (A) and VRN10 (B) loci in the subset of F2 plants with no Hwaseong allele. S, Size marker; H, Hwaseong homozygous; M, Milyang23 homozygous; MN, heterozygous; N, O. minuta homozygous. **Fig S2** Genotype analysis of two extreme phenotype groups to detect markers linked to the additional QTL for seed awning. Polymorphisms of three representative markers, RM252 (A), ID-J2 (B), and RM525 (C), are shown. MM, Milyang23 homozygous; MN, Heterozygous; NN, NIL4/9 homozygous. **Fig S3** Comparison of the awn length in the BC1F2 and BC1F3 populations that are segregating only at qAwn9 (A) and LABA1 (B), respectively. MM, Milyang23 homozygous; MN, heterozygous; NN, O. minuta homozygous on (A) or Hwaseong homozygous on (B). Means labeled with different letters above each box are significantly different among genotypes (Tukey’s test, p < 0.05). n1): number of plants. **Fig S4** Sequence comparison of the LABA1 exon region between parental lines. The black and white boxes indicate exons and UTR regions, respectively. One C-nucleotide deletion (+69 position) was found in the first exon of LABA1 in Milyang23 compared with NIL4/9 and Hwaseong and resulted in a truncated protein with only 55 amino acids compared to 250 amino acids in the wild types. **Fig S5** Manhattan plots of p-values analyzed using a mixed linear model (MLM) for awn development. The red line indicates the threshold. **Fig S6** Gene structure of the OsbZIP76 and SNPs among NIL4/9, Milyang23, and Hwaseong. Black boxes and lines indicate exons and introns, respectively. Red lines indicate nucleotide variation.**Fig S7** Haplotype and allele frequency in OsbZIP76 in the database for big natural populations. (A) Haplotype network of OsbZIP76 constructed using the tool ‘haplotype network analysis’ (http://ricevarmap.ncpgr.cn). Each circle represents a haplotype, and the size is proportionate to the number of accessions in the corresponding haplotype. Branch length represents the genetic distance between two haplotypes. (B) Allele frequency chart of each SNP position in the OsbZIP76 region for all or each subspecies of the 3 K rice accessions (http://snp-seek.irri.org). Ind1, ind2, and ind3 are three groups of indica rice; indx corresponds to other indica varieties, temp is temperate japonica, trop is tropical japonica, japx is other japonica varieties, aus is aus, indx is admixed aus and indica, aro is aromatic and admix is all other unassigned varieties. Red arrows indicate the positions of three missense mutations that we previously detected between NIL4/9, Milyang23, and Hwaseong. **Fig S8** Confirmation of a tropical japonica-specific clustered sequence in the surrounding location of OsbZIP76. Allele frequency chart of each SNP position in Os09g0520100 (A) and Os09g0521300 (B) genes for all or tropical japonica of the 3 K rice accessions (http://snp-seek.irri.org). Red circles indicate the positions of tropical japonica-specific alleles.

**Additional file 2**: **Table S1** List of molecular markers used for QTL analysis and fine mapping.**Table S2** Passport information of 137 rice accessions (KRICE_CORE set). **Table S3** Summary of the whole-genome sequencing result for the parental lines. **Table S4** List of TASUKE 36 O. rufipogon accessions (https://rapdb.dna.affrc.go.jp). **Table S5** Summary information of significant associations detected by GWAS. **Table S6** Average awn lengths (mm) and standard deviations observed for homozygous genotypes derived from critical recombinant plants. **Table S7** List of annotated genes in the 199-kb target region of qAwn9.**Table S8** Summary of variant identification and annotation of candidate genes.

## Data Availability

All data supporting the conclusions described here are provided in tables and figures.

## Abbreviations

bHLH: Basic helix–loop–helix
bZIP: Basic leucine zipper
CTAB: Cetyl trimethy ammonium bromide
DNA: Deoxyribonucleic acid
GWAS: Genome-wide association study
InDels: Insertions/deletions
MLM: Mixed linear model
NIL: Near-isogenic line
PCR: Polymerase chain reactions
QTLs: Quantitative trait loci
SNPs: Single nucleotide polymorphisms
SSR: Simple sequence repeats
