# Supplementary material for: Characterization of Domestication Loci Associated with Awn Development in Rice
Source: Rice (N Y). 2022 Nov 30;15:61. doi: 10.1186/s12284-022-00607-y (PMC9712879; doi:10.1186/s12284-022-00607-y)
Supplement: Supplementary file 1 — Additional file 1: Fig S1 Confirmation of the O. minuta segment in the NIL4/9 background. The O. minuta and Milyang23 alleles were segregated at the RM24663 (A) and VRN10 (B) loci in the subset of F2 plants with no Hwaseong allele. S, Size marker; H, Hwaseong homozygous; M, Milyang23 homozygous; MN, heterozygous; N, O. minuta homozygous. Fig S2 Genotype analysis of two extreme phenotype groups to detect markers linked to the additional QTL for seed awning. Polymorphisms of three representative markers, RM252 (A), ID-J2 (B), and RM525 (C), are shown. MM, Milyang23 homozygous; MN, Heterozygous; NN, NIL4/9 homozygous. Fig S3 Comparison of the awn length in the BC1F2 and BC1F3 populations that are segregating only at qAwn9 (A) and LABA1 (B), respectively. MM, Milyang23 homozygous; MN, heterozygous; NN, O. minuta homozygous on (A) or Hwaseong homozygous on (B). Means labeled with different letters above each box are significantly different among genotypes (Tukey’s test, p < 0.05). n1): number of plants. Fig S4 Sequence comparison of the LABA1 exon region between parental lines. The black and white boxes indicate exons and UTR regions, respectively. One C-nucleotide deletion (+69 position) was found in the first exon of LABA1 in Milyang23 compared with NIL4/9 and Hwaseong and resulted in a truncated protein with only 55 amino acids compared to 250 amino acids in the wild types. Fig S5 Manhattan plots of p-values analyzed using a mixed linear model (MLM) for awn development. The red line indicates the threshold. Fig S6 Gene structure of the OsbZIP76 and SNPs among NIL4/9, Milyang23, and Hwaseong. Black boxes and lines indicate exons and introns, respectively. Red lines indicate nucleotide variation.Fig S7 Haplotype and allele frequency in OsbZIP76 in the database for big natural populations. (A) Haplotype network of OsbZIP76 constructed using the tool ‘haplotype network analysis’ (http://ricevarmap.ncpgr.cn). Each circle represents a haplotype, and the size is proportionat [file 12284_2022_607_MOESM1_ESM.pdf]

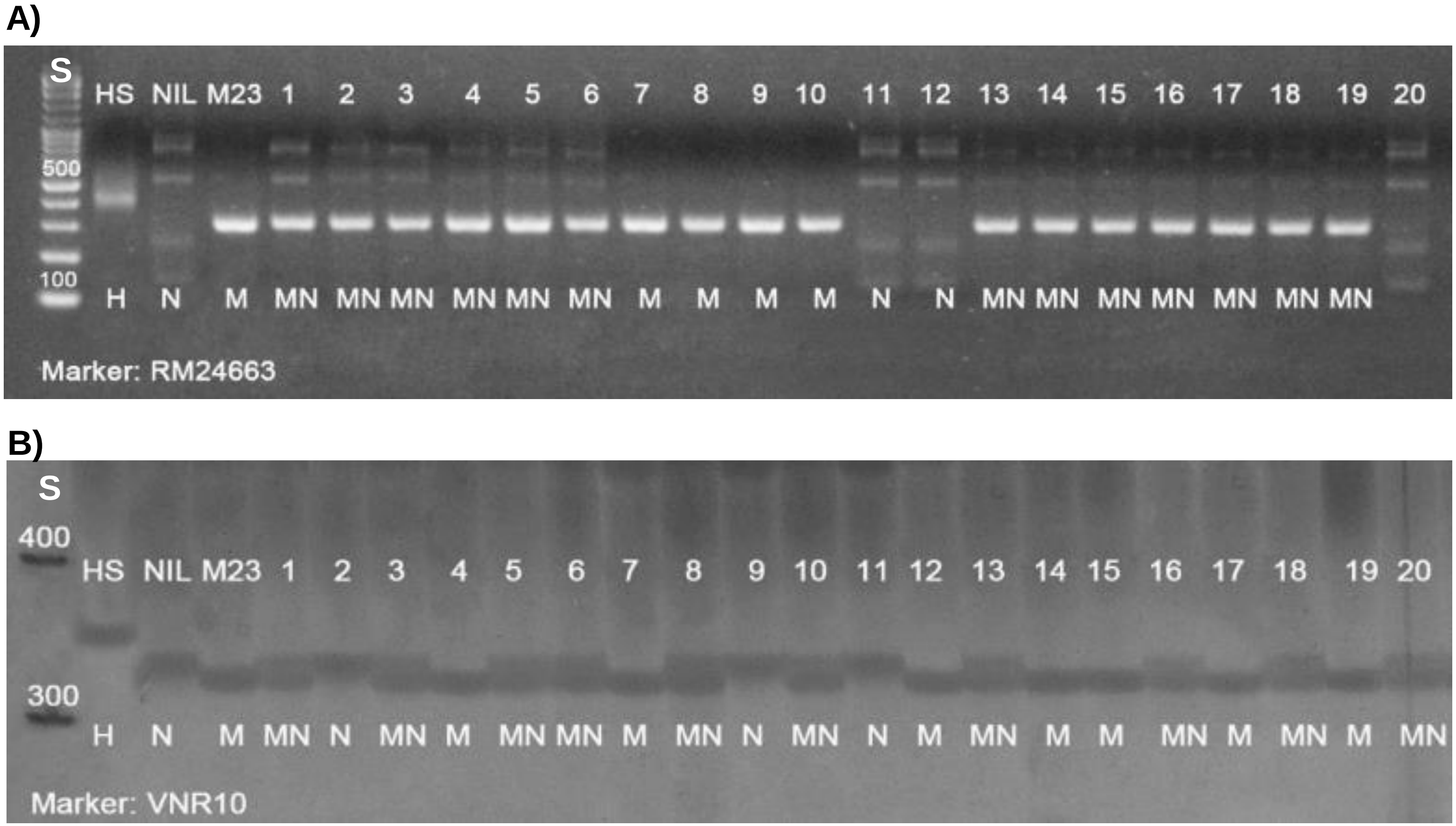

Figure S1. Confirmation of the *O. minuta* segment in the NIL4/9 background. The *O. minuta* and Milyang23 alleles were segregated at the RM24663 (A) and VRN10 (B) loci in the subset of F_2 plants with no Hwaseong allele. S, Size marker; H, Hwaseong homozygous; M, Milyang23 homozygous; MN, heterozygous; N, *O. minuta* homozygous.

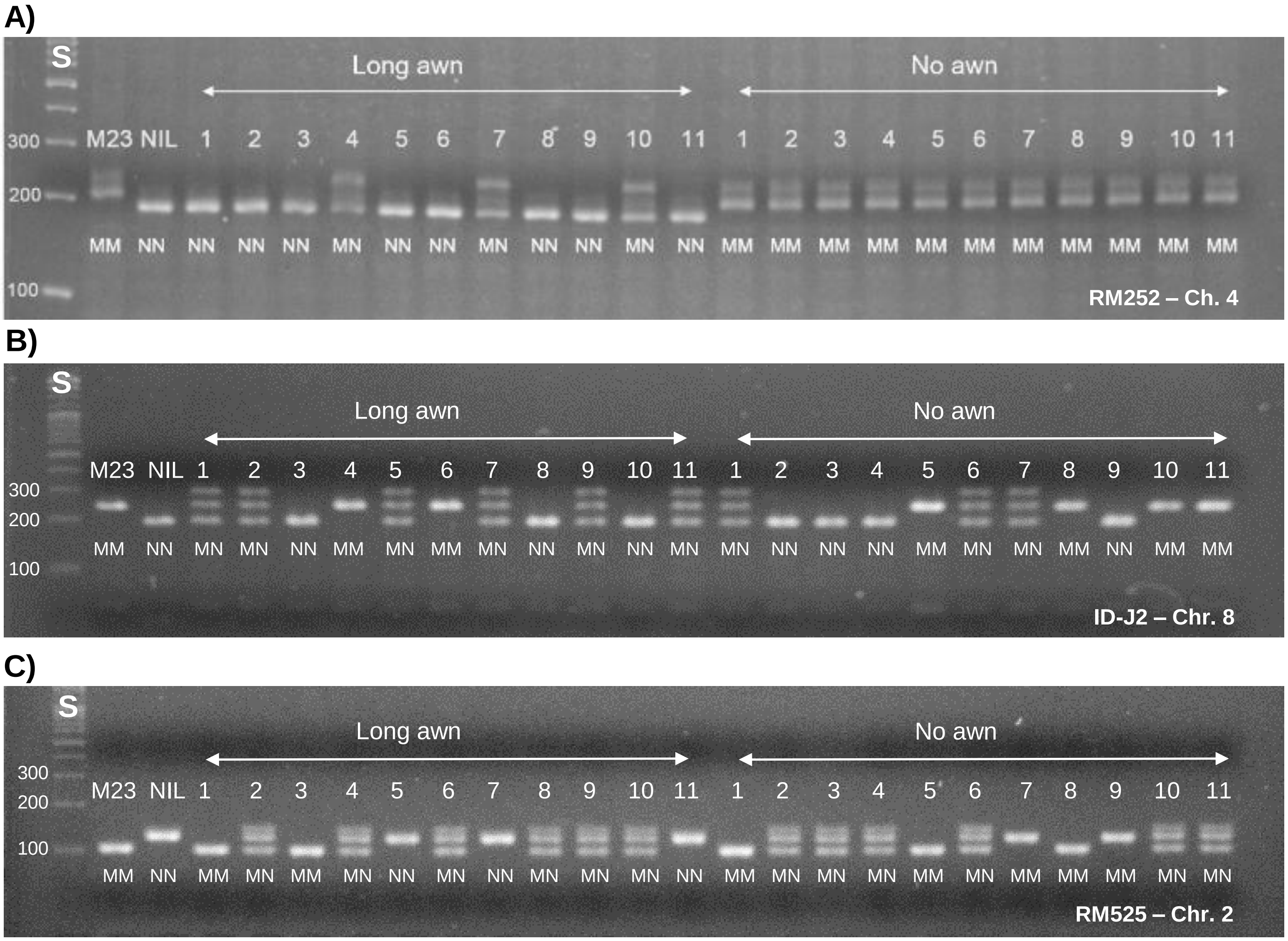

Figure S2. Genotype analysis of two extreme phenotype groups to detect markers linked to the additional QTL for seed awning. Polymorphisms of three representative markers, RM252 (A), ID-J2 (B), and RM525 (C), are shown. MM, Milyang23 homozygous; MN, Heterozygous; NN, NIL4/9 homozygous.

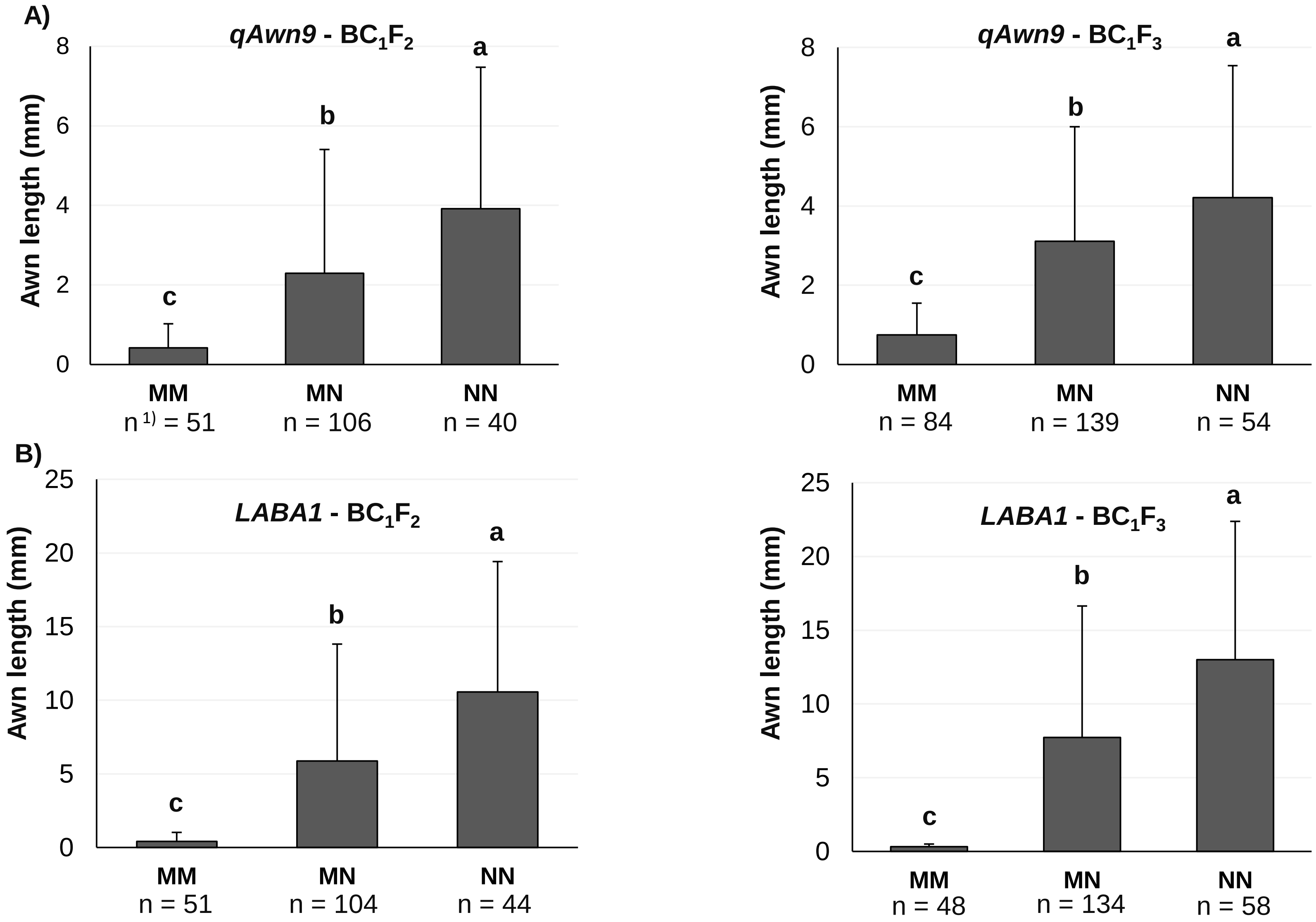

Figure S3. Comparison of the awn length in the BC_1F_2 and BC_1F_3 populations that are segregating only at *qAwn9* (A) and *LABA1* (B), respectively. MM, Milyang23 homozygous; MN, heterozygous; NN, *O. minuta* homozygous on (A) or Hwaseong homozygous on (B). Means labeled with different letters above each box are significantly different among genotypes (Tukey's test, $P < 0.05$). n[1)]: number of plants

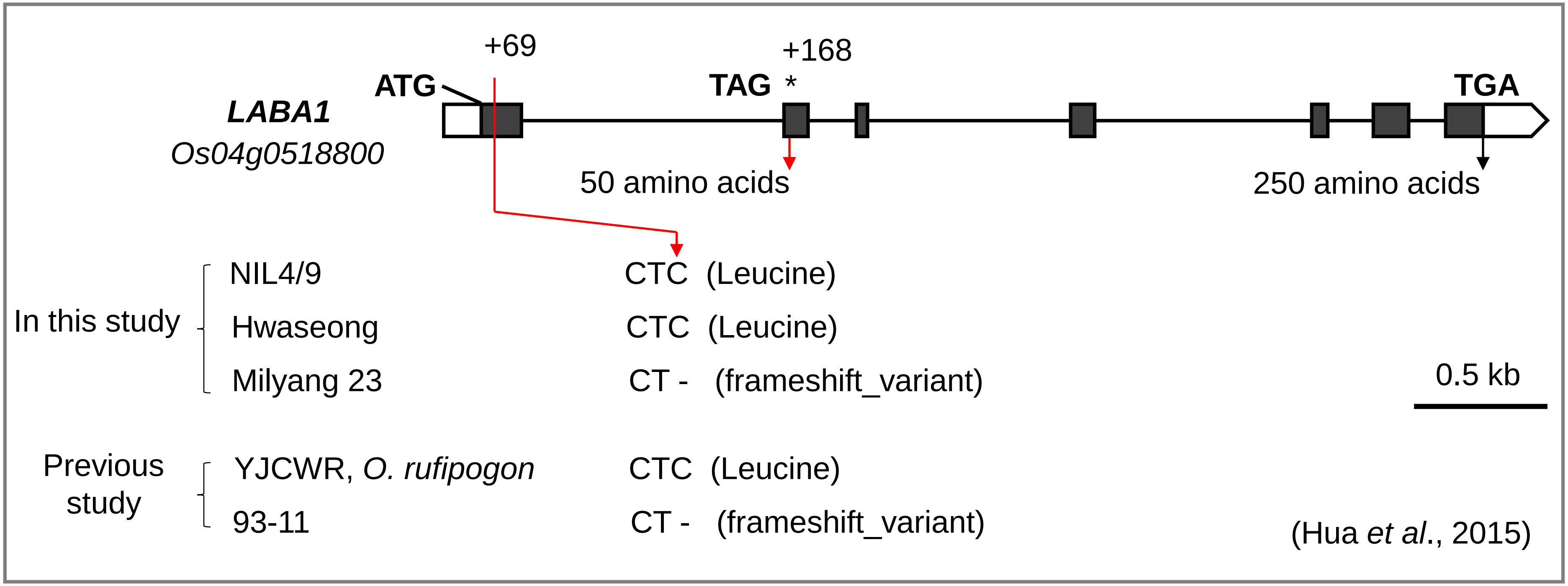

Figure S4. Sequence comparison of the *LABA1* exon region between parental lines. The black and white boxes indicate exons and UTR regions, respectively. One C-nucleotide deletion (+69 position) was found in the first exon of *LABA1* in Milyang23 compared with NIL4/9 and Hwaseong and resulted in a truncated protein with only 55 amino acids compared to 250 amino acids in the wild types.

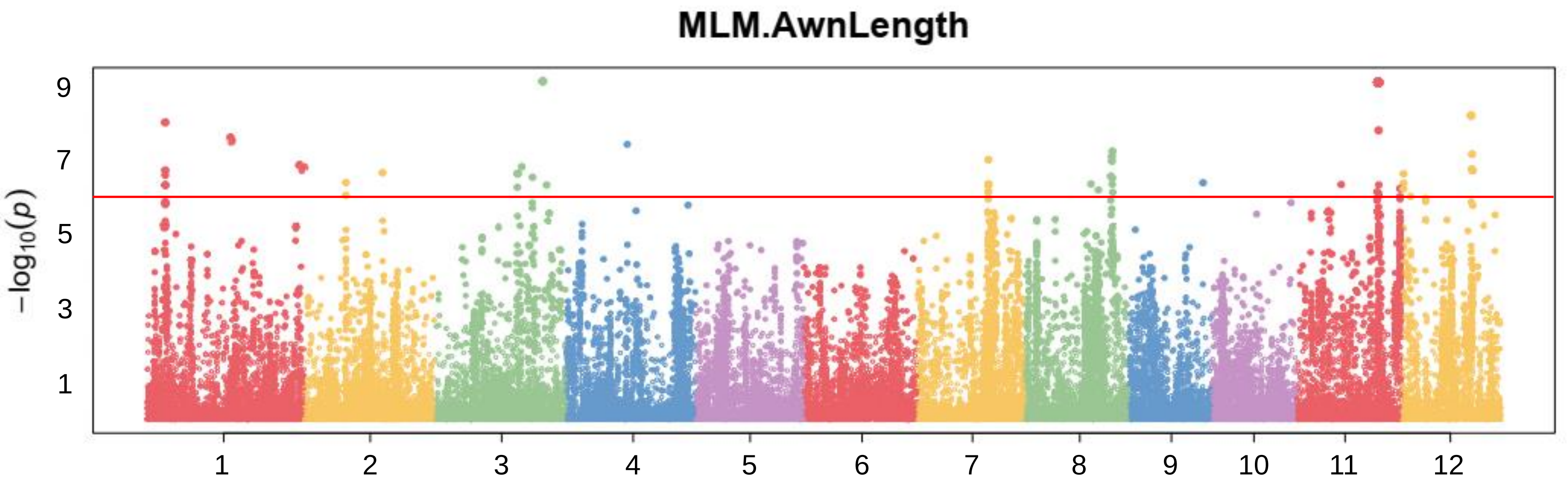

Figure S5. Manhattan plots of p-values analyzed using a mixed linear model (MLM) for awn development. The red line indicates the threshold.

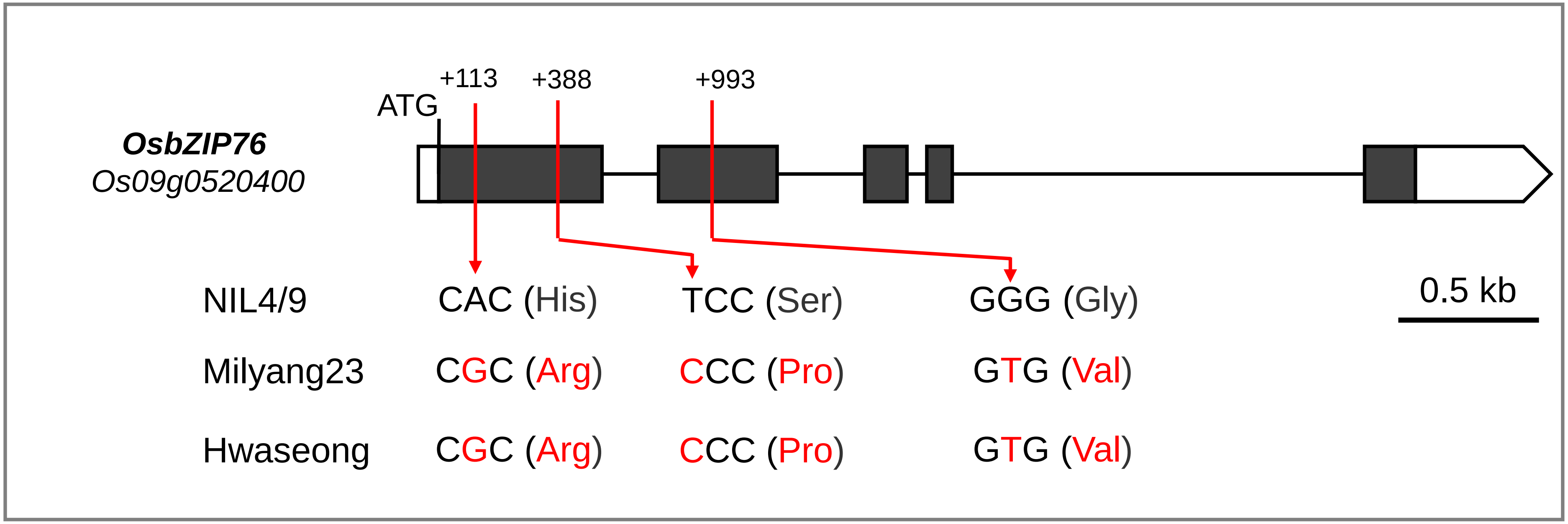

Figure S6. Gene structure of the *OsbZIP76* and SNPs among NIL4/9, Milyang23, and Hwaseong. Black boxes and lines indicate exons and introns, respectively. Red lines indicate nucleotide variation.

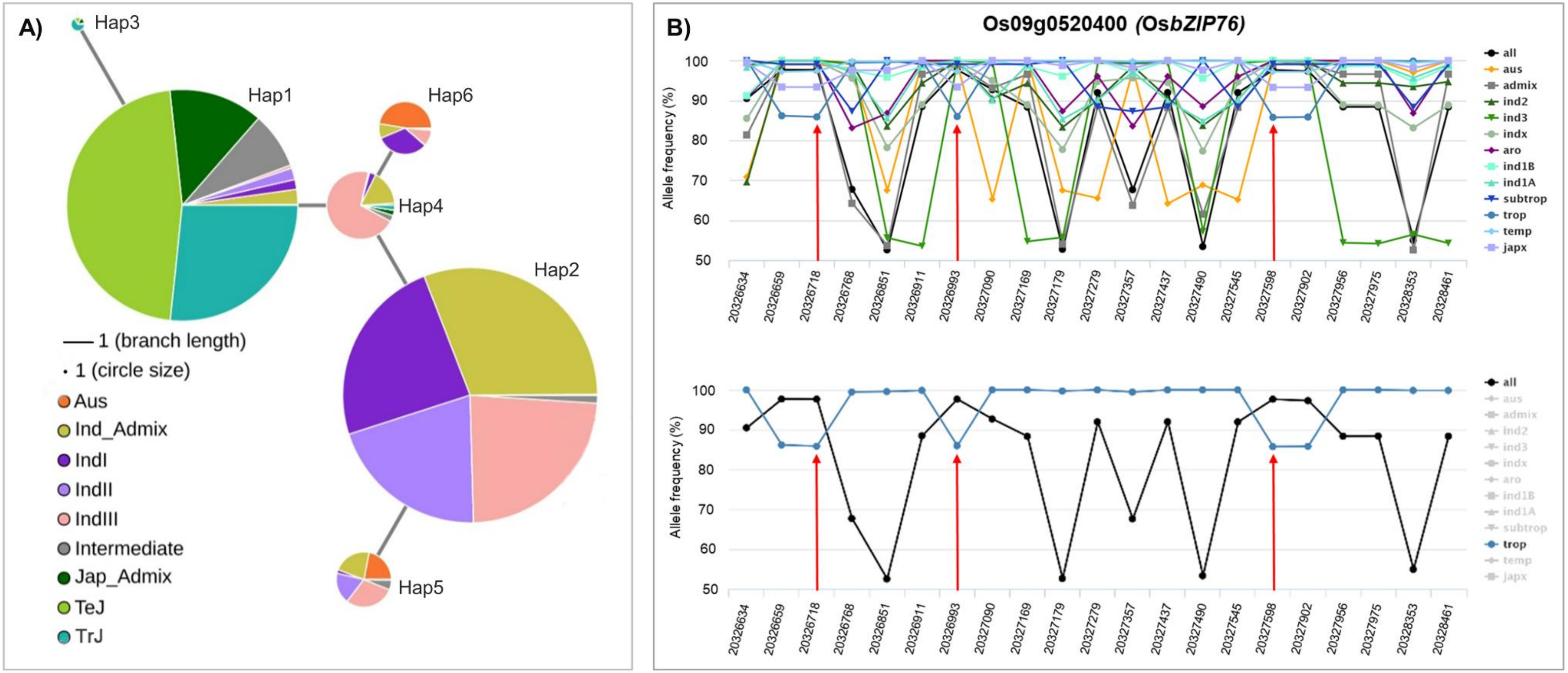

Figure S7. Haplotype and allele frequency in Os*bZIP76* in the database for big natural populations. (A) Haplotype network of Os*bZIP76* constructed using the tool 'haplotype network analysis' (http://ricevarmap.ncpgr.cn). Each circle represents a haplotype, and the size is proportionate to the number of accessions in the corresponding haplotype. Branch length represents the genetic distance between two haplotypes. (B) Allele frequency chart of each SNP position in the Os*bZIP76* region for all or each subspecies of the 3 K rice accessions (http://snp-seek.irri.org). Ind1, ind2, and ind3 are three groups of *indica* rice; indx corresponds to other *indica* varieties, temp is temperate *japonica*, trop is tropical *japonica*, japx is other *japonica* varieties, aus is aus, indx is admixed aus and *indica*, aro is aromatic and admix is all other unassigned varieties. Red arrows indicate the positions of three missense mutations that we previously detected between NIL4/9, Milyang23, and Hwaseong.

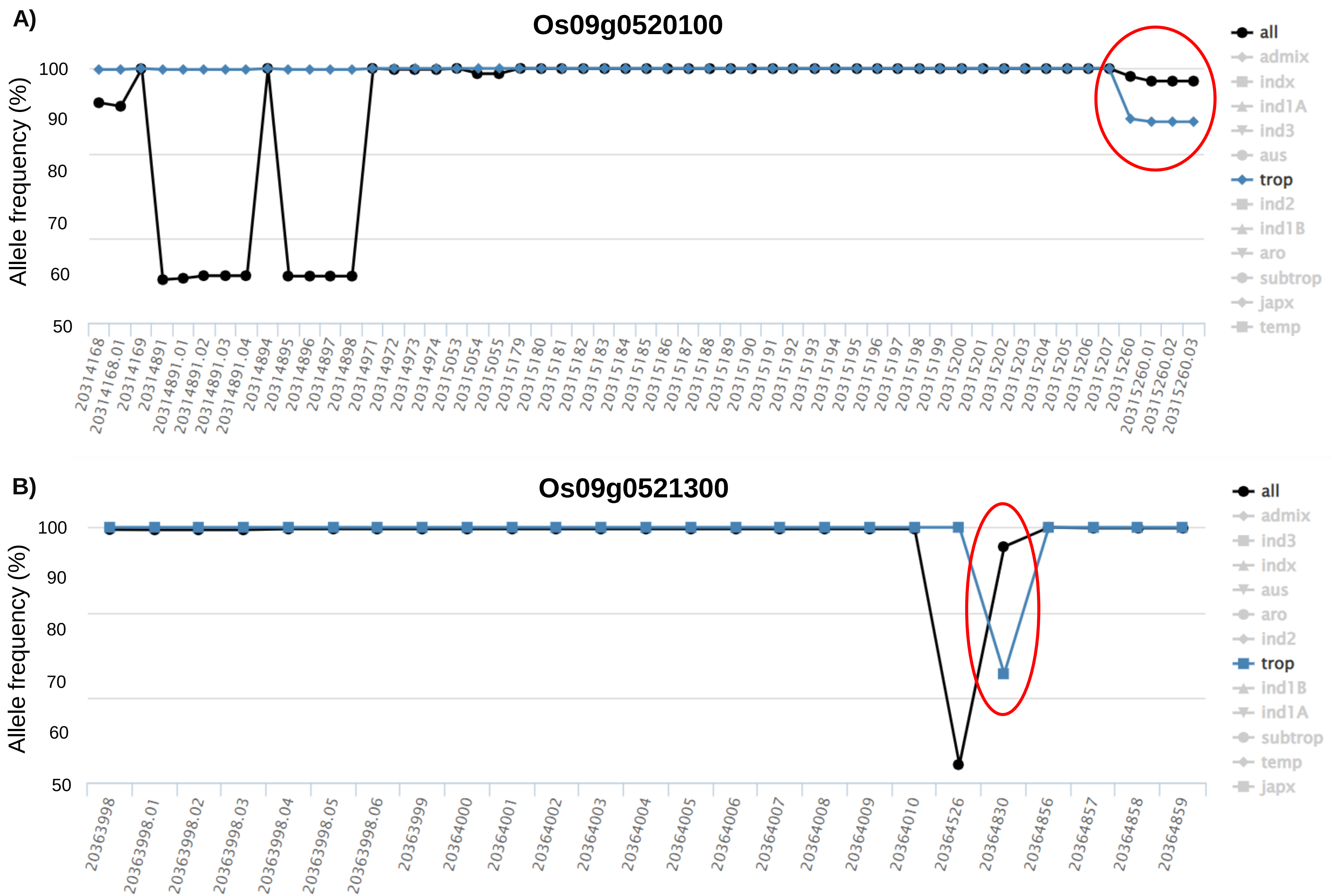

Figure S8. Confirmation of a tropical *japonica*-specific clustered sequence in the surrounding location of *OsbZIP76*. Allele frequency chart of each SNP position in Os09g0520100 (A) and Os09g0521300 (B) genes for all or tropical *japonica* of the 3 K rice accessions (http://snp-seek.irri.org). Red circles indicate the positions of tropical *japonica*-specific alleles.
